# Improving Human Plateaued Motor Skill with Somatic Stimulation

**DOI:** 10.1371/journal.pone.0025670

**Published:** 2011-10-04

**Authors:** Shintaro Uehara, Isao Nambu, Saeka Tomatsu, Jongho Lee, Shinji Kakei, Eiichi Naito

**Affiliations:** 1 Brain ICT Laboratory, National Institute of Information and Communications Technology, Kyoto, Japan; 2 Graduate School of Human and Environmental Studies, Kyoto University, Kyoto, Japan; 3 The Japan Society for the Promotion of Science, Tokyo, Japan; 4 Tokyo Metropolitan Institute of Medical Science, Tokyo, Japan; 5 Center for Information and Neural Networks, Osaka, Japan; 6 Graduate School of Medicine, Osaka University, Osaka, Japan; Katholieke Universiteit Leuven, Belgium

## Abstract

Procedural motor learning includes a period when no substantial gain in performance improvement is obtained even with repeated, daily practice. Prompted by the potential benefit of high-frequency transcutaneous electrical stimulation, we examined if the stimulation to the hand reduces redundant motor activity that likely exists in an acquired hand motor skill, so as to further upgrade stable motor performance. Healthy participants were trained until their motor performance of continuously rotating two balls in the palm of their right hand became stable. In the series of experiments, they repeated a trial performing this cyclic rotation as many times as possible in 15 s. In trials where we applied the stimulation to the relaxed thumb before they initiated the task, most reported that their movements became smoother and they could perform the movements at a higher cycle compared to the control trials. This was not possible when the dorsal side of the wrist was stimulated. The performance improvement was associated with reduction of amplitude of finger displacement, which was consistently observed irrespective of the task demands. Importantly, this kinematic change occurred without being noticed by the participants, and their intentional changes of motor strategies (reducing amplitude of finger displacement) never improved the performance. Moreover, the performance never spontaneously improved during one-week training without stimulation, whereas the improvement in association with stimulation was consistently observed across days during training on another week combined with the stimulation. The improved effect obtained in stimulation trials on one day partially carried over to the next day, thereby promoting daily improvement of plateaued performance, which could not be unlocked by the first-week intensive training. This study demonstrated the possibility of effectively improving a plateaued motor skill, and pre-movement somatic stimulation driving this behavioral change.

## Introduction

Humans have great ability to learn different types of motor skills, yet in humans and, even in animals, motor learning includes a period when no substantial gain in performance improvement is observed, even with repeated, daily practice [1], [2]. This behavioral phenomenon is generally called a “learning plateau” or “performance asymptote,” which is frequently experienced by people in late-stage motor learning when performance reaches a certain level of stability. Once the learning plateaus, further performance upgrading is normally difficult with simple repetition of physical training, and no ingenious attempts have been made to overcome the behavioral plateau.

If one considers the empirical knowledge that very well trained athletes, musicians and craftsmen can still improve their performance by practicing, an apparent behavioral plateau can be a sub-optimal state in motor learning. Since optimal performance of a skilled motor task seems to be achieved through processes in which the nervous system controls redundancy in task performance [3], one may expect to facilitate further optimization of seemingly plateaued motor performance as long as the performance contains nonessential components in its control, which can somehow be reduced.

High-frequency transcutaneous electrical stimulation to human hand has been shown to have an effect of reducing excessive motor activity, such as muscle hypertonia, after stimulation by modulating sensory-motor neuronal states [4]. In the present study, we evaluated potential benefits of high-frequency electrical stimulation in the control of a hand motor skill by testing whether the stimulation may reduce nonessential motor activity potentially existing in an acquired motor skill, so as to upgrade the stabilized motor performance.

**Figure 1:**
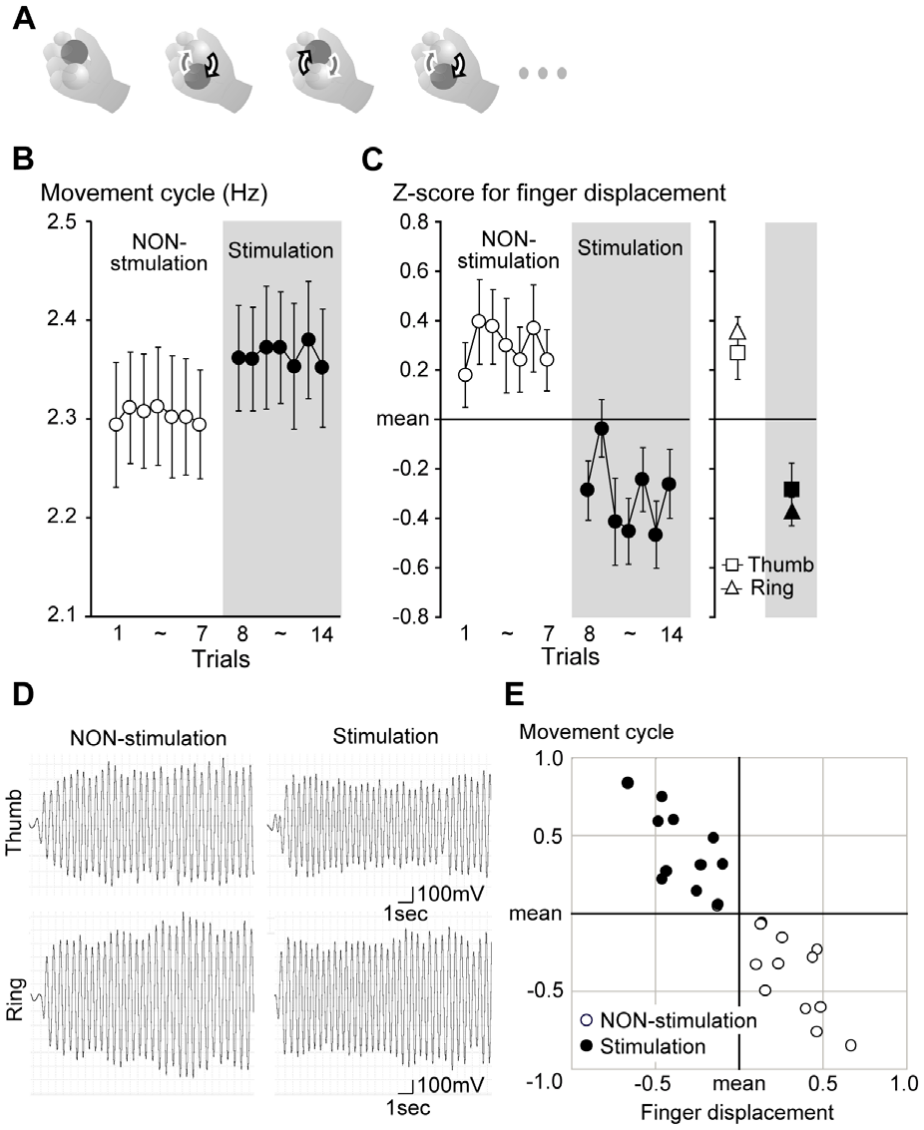
Motor task (A) and results from experiment 1 (B–E). (B) Average movement cycle across participants (*y* axis) in sequence of trials (*x* axis). White and black dots represent data in NON-stimulation and stimulation trials, respectively. Small bars indicate standard error of means across participants (SEM). (C) Average z-scores for amplitude of finger displacement per cycle across participants (*y* axis) in sequence of trials (*x* axis). For each participant, z-score was first calculated by averaging data from thumb and ring finger. White and black dots represent data in NON-stimulation and stimulation trials, respectively. In the right panel, data from each finger (squares for thumb; triangles for ring finger) are shown. Open symbols indicate grand average data for all NON-stimulation trials of all participants, and filled ones represent those for stimulation trials. (D) Representative examples of finger displacement in NON-stimulation (left) and stimulation (right) trials obtained from a participant. Top panels represent data from thumb and bottom panel represents data from ring finger. (E) Relationship between z-scores for movement cycle (*y* axis) and for amplitude of finger displacement per cycle (*x* axis) across participants. For the finger displacement, we used the average z-score from the two fingers. The movement cycle was also converted to a z-score in the same way as described in the text. Each white dot represents the average z-score for NON-stimulation trials for each participant, and each black dot represents that for individual stimulation trials.

We applied high-frequency stimulation to the relaxed thumb just before participants initiated a motor task of skillful manipulation of external objects with the fingers, involving cyclic rotation of two balls in the palm of the hand [5], [6]. Before we began the series of experiments, the participants were trained until their performance became stabilized (see Figure 1B and Figure S1). In the experiments (except for one control experiment), they repeated a trial wherein they performed the cyclic rotation as many times as possible in 15 s with certain inter-trial-intervals (ITI). In the first experiment, we applied the stimulation to the thumb during the ITI before a trial, which was repeated in half of the trials. We explored behavioral changes in these trials by comparing with the performance in control trials where no stimulation was applied (Experiment 1). Here, to evaluate the effectiveness of the thenar stimulation, we also examined behavioral changes when we administered the stimulation to the dorsal surface of the wrist joint (instead of the thumb) in the same experimental design. Since we found that the thenar stimulation seemed to be effective for generating behavioral changes, we verified this effects by conducting control experiments (Experiment 2). Finally, we also examined the stimulation effects when we applied the thenar stimulation during daily training (Experiment 3). Through these experiments, we evaluated the effectiveness of high-frequency electrical somatic stimulation on human plateaued motor control and learning.

## Materials and Methods

### General

#### Participants

A total of 12 (10 male and 2 female; aged 22–41) right-handed volunteers participated in the series of experiments. We examined their handedness using the Oldfield questionnaire [7], and confirmed that they were all right-handers. The laterality quotient ranged from 86.7 to 100. The Ethical Committee of the National Institute of Communication and Technology (NICT) approved the study. All participants provided written informed consent. The experiment was performed according to the principles and guidelines of the Declaration of Helsinki (1975).

#### Motor task and training

The participants continuously rotated two balls (diameter 35 mm; 80 g each) in the palm of the right hand as many times as possible, with eyes closed (Figure 1A). This task requires smooth coordination of all finger movements, with little aid from verbal-cognitive factors; thus, the brain must implicitly acquire a sequential pattern of combined finger movements by optimizing their displacement and timing [6].

In order to stabilize motor performance before we started the series of experiments, all participants were intensively trained for at least three days (30 min per day) in the experiment room, since we knew from our previous experiments that the motor performance tended to stabilize from the third day of daily practice. Since we wanted them to thoroughly learn the task, we also allowed them to practice outside of the experimental room (which was strictly prohibited during the two-week training; see below). Before starting the first experiment, we confirmed that all participants could rotate the balls at their stabilized cycles with no significant improvement within trials (seven 15-s trials with ITI of 50 s; see supporting text S1 and Figure S1). Thus, the experiments were conducted in a situation where robust performance improvement was difficult to be naturally seen within the trials of an experimental day.

#### Electrical stimulation

To test the effect of electrical stimulation on motor performance, we applied it to the right thumb during the pre-trial ITI, and repeated this in successive trial series. Due to synergistic organization of the finger muscles, we stimulated the thumb, but expected derivative benefits in the control of other fingers [8], [9]. Each stimulation during the ITI lasted for 90 s. We expected this short-period stimulation to be effective, because even short-period (less than 1 min), high-frequency somatosensory stimulation to a relaxed limb is capable of modulating sensory-motor neuronal states [10], [11]. Electrical stimulation was delivered using a portable constant current stimulator (SUN MASSEUR HOT22BL; Marubishi Sangyo, Osaka, Japan), and a pair of electrical patches (Long Life Pad no. 29B2X00003000007, each about 3×2 = 6 cm^2^; OMRON, Kyoto, Japan) was attached to the skin surface on the palm over the *abductor pollicis brevis* (APB) muscle. The anode patch was distally located (DC mode). The stimuli were delivered at 100 Hz with pulse duration of 250 µs. We administered trains of on-off patterned stimulation [i.e., 11-s stimulation with inter-stimulation interval (ISI) of 3 s], with each train as trapezoid-wave stimulation. The intensity was set at just below the motor threshold (MT), which elicits no visible thumb movements nor muscle twitch. We used high-frequency and low-intensity stimulation because this type of stimulation is known to be capable of benefiting subsequent motor behaviors by reducing excessive motor activity [4]. The intensity was determined prior to the start of the experiment for each participant. The stimulus intensity ranged from 2.6 to 8.7 mA across all participants in all experiments. The average intensity was 5.5 mA. The thenar stimulation generally produced both tingling and muscle-stimulation sensations without producing any overt muscle responses. This is probably because sensory fibers with larger diameter are more sensitive to the electrical stimulation than efferent fibers [12].

Finally, to confirm the effectiveness of the thenar stimulation, we also evaluated the change of motor performance when we administered identical (same intensity, pattern and stimulus length) stimulation to the dorsal side of the wrist by attaching the patches to the skin surface over the *processus styloideus ulnae and radius* (see below). We also checked possible placebo effects from the thenar stimulation (see also below).

#### Task procedure

The trained, blindfolded participants were asked to continuously rotate two balls as many times as possible in 15 s (1 trial), and repeated the trial 14 times with an inter-trial interval (ITI) of 120 s. The trial duration, number of trial repetitions and ITI were consistent in all experiments. In all experiments, except for the constant rotation task (see below), the task always required that participants exert maximum effort in rotating the balls as many times as possible in each trial (maximum rotation task). The start and end of the trial were signaled by computer sounds, and start timing was counted from 3 s before each trial.

Each participant performed the task while seated with his or her tested arm resting on a table in the supine position, with the forearm supported with a cushion. The participants were allowed to move only their fingers without moving the forearm. During ITI, the participants were requested to immobilize and relax their hands to avoid effects of muscular thixotropy [13].

#### Behavioral measurement

To measure the motor performance, small sensors (0.2 g; Vibration Pickup Model 2351A; Showa Sokki Corporation, Tokyo, Japan) were mounted to the nails of the thumb and ring finger. The signals were amplified (Vibration Meter Model 1607A; Showa Sokki Corporation, Tokyo, Japan) and recorded in the displacement measurement mode. During the cyclic finger movements, the sensor provided a sinusoidal-like waveform, of which positive and negative peaks respectively represented flexion or extension of finger movements. Thus, the data (Figure 1D) contained information regarding the movement cycle as well as the amplitude of each finger displacement (extension-flexion) per cycle.

The kinematic signals were recorded on a PC for later offline analyses via an A/D converter (PowerLab 16/30; ADInstruments Japan Inc., Nagoya, Japan). The data were sampled at 2 kHz. The converter also received event signals that indicated the onset and offset of trials from the computer. The duration of each trial was defined by these event signals. In the analysis, the data obtained from the sensors were first band-pass filtered (1–4 Hz).

#### Evaluation of movement cycle

The movement cycle representing the number of rotations per trial was analyzed in all experiments. In each trial of each participant, we measured the duration of each cycle, which was defined as the time between two adjacent positive peaks in the data provided by the sensor. This was performed separately for the thumb and ring finger. We calculated the mean duration of the cycles for each trial, which was ultimately converted to hertz. Since the movement cycles obtained from the two fingers were highly similar, we defined the cycle based on the data obtained from the thumb.

#### Evaluation of amplitude of finger displacement

The amplitude of finger displacement per cycle was analyzed in experiments 1 and 2 (see below). The sensor provided quite variable signals, which depended on the location of the sensor attached on the finger nail and the pressure from the adhesive tape that attached the sensor to the finger. This feature of the sensor only allowed us to evaluate the changes in behavior within a session in one experimental day, and the comparison across experimental days was quite unreliable. Therefore, we did not show the amplitude of finger displacement in experiment 3 where we measured behavioral change across days (see below).

The amplitude of finger displacement was defined as the difference between values of the positive and subsequent negative peaks in each cycle. This was performed separately for the thumb and ring finger. In this analysis, we excluded the first two cycles in each trial because these data often reflected the initial state of performance, which might not have stabilized, as indicated by the relatively small amplitude of displacement (Figure 1D). We calculated the mean amplitude of finger displacement per cycle for each trial.

The amplitude varied across participants, probably due to individual differences of physical length of fingers and/or of motor strategy to perform the task. Thus, the mean amplitude in each trial was converted to a z-score on the basis of the data obtained from all trials for each participant. This was performed separately for the thumb and ring finger.

### Experiment 1: Evaluation of thenar stimulation effect in maximum rotation task

The 12 trained, blindfolded participants performed the maximum rotation task. In experiment 1, no stimulation was delivered in the first seven (NON-stimulation) trials, while the thenar stimulation was delivered in the remaining (stimulation) trials. In stimulation trials, the patches were placed on the thumb immediately after the previous trial (within 5 s). After completing the stimulation (90 s), the patches were removed, and the task was executed immediately after completion of ITI. Thus, the task was initiated about 20 s after cessation of stimulation. These stimulation procedures were consistent across experiments where we administered electrical stimulation. As a warm-up, they rotated the balls in several trials before the experiment.

#### Analysis

For the movement cycle, we calculated the mean movement cycle (hertz) in each trial across participants (Figure 1B). Likewise, we calculated the mean z-score for the amplitude of finger displacement per cycle in each trial across participants (Figure 1C). Since we found the same trend (reduction of amplitude of displacement) in the thumb and ring finger in stimulation trials, for the following statistical evaluation we averaged the data obtained from the two fingers for each participant.

To statistically evaluate behavioral changes in stimulation trials, a two-factorial [stimulation or NON-stimulation (2) × trials for each (7)] analysis of variance (ANOVA; repeated measurement; n = 12) was performed for the movement cycle and the z-score of the amplitude (Figures 1B, C). To evaluate the fast-acting effect (see Results) we performed a paired-t test, both for the movement cycle and the amplitude, by comparing the data obtained from the last NON-stimulation (7th) trial and first stimulation (8th) trial.

#### Evaluation of wrist or sham stimulation effect

We found the thenar stimulation changed the motor performance (Figures 1B, C). We conducted these investigations to confirm the importance of thenar stimulation and rebuff the possible involvement of placebo effects from the stimulation.

Seven of 12 participants were recruited and performed the same maximum rotation task (7 NON-stimulation trials and 7 stimulation trials). However, in stimulation trials the patches were placed over the skin surface over the *processus styloideus ulnae and radius (wrist stimulation)*. Thus, in this experiment, we stimulated the dorsal side of the wrist joint, instead of the thumb. We first determined the stimulus intensity (just below MT; see above) for the thumb before the experiment, and used this intensity in the wrist stimulation trials. Details of the stimulation procedure were identical in the thenar stimulation trials. During the wrist stimulation, all participants reported being aware of the tingling sensation in the wrist, but the stimulation never evoked visible muscular contraction around the wrist. Thus, the wrist stimulation likely attracted the participant's attention to the hand, similar to the case of the thenar stimulation, and could be an experimental maneuver to control involvement of attentional factors in behavioral changes.

We also investigated the sham stimulation effect in the 7 participants. In this investigation, we delivered the same electrical stimulation to the skin surface over the thenar muscle but only at the beginning (only one on-off patterned cycle) during each ITI (120 s) of last 7 trials in 14 trials. The stimulation was terminated without informing the participants. This could be another experimental maneuver to see if the performance change caused by the thenar stimulation is merely attributed to the placebo effects from the stimulation.

In both evaluation of wrist or sham stimulation effect, the same two-factorial [stimulation or NON-stimulation (2) × trials for each (7)] ANOVA was performed to statistically evaluate the behavioral change.

### Experiment 2: Control experiments

#### Constant rotation task

In experiment 1, we found the reduction of amplitude of finger displacement in stimulation trials (Figures 1C, D). We conducted a control experiment to confirm if this would also occur in stimulation trials when the task required constant rotation at a regular pace. This experiment was performed about 15 min after completion of experiment 1.

The same 12 participants performed the same NON-stimulation and stimulation trials (7 trials each), but at a regular pace of 80% of their maximum movement cycle (constant rotation task). This pace was determined for each participant, and was 80% of the average movement cycle obtained in the seven NON-stimulation trials of experiment 1. Thus, the task became easier and less demanding, and required no particular motivation in order to exert maximum effort. Each cyclic movement was paced by an auditory cue, with which the participants had to continuously rotate the balls in synchronization. As practice, the participants performed several trials to get used to the auditory pace.

In this experiment, by simultaneously recording electromyogram (EMG) from the APB muscle during the task, we also checked if possible reduction of amplitude of finger displacement may accompany reduction of its associated muscular activity. We avoided EMG recording in other experiments where the maximum rotation task was required, to prevent an electrode from disrupting the participant's best performance. EMG was recorded by using an electrode (BA-U410; Nihon Santeku, Osaka, Japan) attached to the skin surface over the APB muscle. The electrode was placed on the skin over the muscle belly, which was identified by palpation during associated thumb movement. During thenar stimulation, small electrical patches were placed just beside the electrode. The EMG activities were amplified 1,000 times with an amplifier (BA1104m; Nihon Santeku, Osaka) and stored on the same recording system (see above). The EMG data were high-pass filtered (50 Hz) and rectified. First, the integrated EMG for each cycle was calculated, and the mean integrated EMG per cycle was calculated for each trial for each participant. The general procedures of EMG analysis are described elsewhere [6].

Both the amplitude of thumb displacement per cycle and the integrated EMG from the APB muscle were analyzed, and z-scored in the same way as described above. The same two-factorial [stimulation or NON-stimulation (2) × trials for each (7)] ANOVA was performed for the z-scores for the amplitude of thumb displacement and the integrated EMG, respectively.

#### Effect of volitional effort in motor performance

In experiment 1, the reduction of amplitude of finger displacement was associated with the increase of movement cycle in stimulation trials (Figure 1E). We conducted this experiment to check if the performance improvement (increase of movement cycle) could be achieved by the participant's volitional effort to reduce amplitude of finger displacement.

Seven of 12 participants were recruited, and performed the same maximum rotation task without receiving thenar stimulation. In the first seven trials, they performed the task given no specific instructions about motor strategy. However, during the ITI before the 8th trial, a special instruction to reduce the size of their finger movements was given, and they had to obey this during their performance of the remaining trials. To statistically evaluate the behavioral changes in this experiment, the two-factorial [before or after instruction (2) × trials for each (7)] ANOVA was performed for the movement cycle and for the z-scores for the amplitude of thumb displacement.

### Experiment 3: Evaluation of thenar stimulation effects during daily training (two-week training)

Finally, we examined the thenar stimulation effects when we repeatedly applied this during daily training. Ten of 12 participants performed the maximum rotation task (14 trials) on each of 10 days. In the five successive days of the first week (NON-stimulation training), no stimulation was administered in any of the trials. This was performed to see the natural change of motor performance when the participants simply repeated the training, and thus could be a control when evaluating stimulation effects. In contrast, in the five days of the second week (stimulation training) we administered the thenar stimulation before each of the last 9 (6th–14th) trials. Thus, the task consisted of 5 NON-stimulation and 9 stimulation trials. We used this design to check if the stimulation effects (fast-acting and retention; see Results) could be consistently observed when we change the timing of stimulation in the sequence of trials, and if the improved performance in stimulation trials somehow carried over to the next day. The design allowed us to evaluate the participants' baseline performance in the 1st-5th NON-stimulation trials on each experimental day, under no influence from the preceding stimulation. Then, we examined the daily change of their baseline performance by comparing the performances in the 1st-5th NON-stimulation trials between during NON-stimulation and stimulation training.

The task was conducted at the same time of day on each day to avoid a circadian factor affecting motor performance [14]. Importantly, the participants were instructed not to practice this task outside the experiments for two weeks.

For statistical evaluation of behavioral change, we separately calculated the mean movement cycle for the 1st-5th trials and for the 6th–14th trials on each training day, and performed three-way [stimulation or NON-stimulation training (2) × 1st-5th trials or 6th–14th trials (2) × days (5)] ANOVA (n = 10). Since we observed that the performance in the 1st-5th NON-stimulation trials improved only during stimulation training, we also performed two-way [stimulation or NON-stimulation training (2) × days (5)] ANOVA by focusing on the baseline performance. Finally, to demonstrate the individual difference in the change of baseline performance across training days, we calculated the improvement ratio of the movement cycle on the last (5th or 10th) day in each week compared with that on the first (1st or 6th) day of the corresponding week, which was performed by using the mean movement cycle for the 1st-5th trials on each participant.

## Results

### Experiment 1: Higher-cycle movement became possible and amplitude of finger displacement reduced in thenar stimulation trials of maximum rotation task

Before we started the experiment, we confirmed that all participants could rotate the balls at 2.3±0.06 Hz (average ± standard errors of means), and their performance was stabilized with no significant improvement within 7 trials (see supporting text S1 and Figure S1).

Since the participants were thoroughly trained before the experiment, the movement cycle was also stabilized and showed no increase across the NON-stimulation trials (Figure 1B), even though they exerted maximum effort. This result was in contrast to the drastic improvement of the movement cycle generally observed in the early stage of learning [6]. Thus, the present finding verified that the experiment was conducted in the learning stage when the participant's performance was stabilized.

In the thenar stimulation trials, however, most of the participants reported that their finger movements became smoother, and they could perform them at a higher cycle compared to the NON-stimulation trials [F(1, 11) = 20.2, p<0.001]. Surprisingly, this became possible immediately in the first stimulation trial (8th trial overall) [t = 4.1, df = 11, p<0.005, in comparison with the last NON-stimulation (7th) trial; Figure 1B]. Thus, the stable performance in the NON-stimulation trials immediately improved with the first application of the stimulation (fast-acting effect). This rapid change of motor behavior may rebuff any possible involvement of natural learning, practicing or warm-up factors in the improvement. Following the fast-acting effect, the participants could continue performing this higher-cycle movement during repetition of the remaining stimulation trials (retention). We confirmed that these behavioral changes were not possible when the stimulation was applied to the dorsal side of the wrist (p = 0.4), nor when the stimulation was applied to the thenar muscle only at the beginning of ITI (p = 0.82).

In the thenar stimulation trials, we also found that the amplitude of finger displacement per cycle was reduced, which was not observed in the wrist stimulation trials (p = 0.3), nor in the sham stimulation trials (p = 0.41). This reduction was observed not only in the stimulated thumb but also in the non-stimulated ring finger (Figures 1C, D), and the amplitude reduction of finger (thumb and ring finger) displacement became significant compared to the NON-stimulation trials [F(1, 11) = 22.7, p<0.001; Figures 1C, D]. The reduction also occurred immediately in the first stimulation trial (t = 2.9, df = 11, p<0.05; Figure 1C; fast-acting effect), which was maintained during repetition of stimulation trials (retention). Importantly, none of the participants explicitly noticed this kinematic change. Thus, it is likely that this change was automatically induced by the thenar stimulation, rather than being caused by participants' intentional and strategic changes (see also below). Hence, the thenar stimulation enabled the participants to increase the movement cycle by reducing (scaling) the kinematic amplitude of each component of the cyclic movements. This indicated that the higher-cycle movement was gained as a favor of promoted efficiency in motor control, rather than at the cost of accuracy in finger movement (Figure 1E).

### Experiment 2: Thenar stimulation reduced amplitude of finger displacement irrespective of task demands, which was difficult to achieve by participant's volition

**Figure 2:**
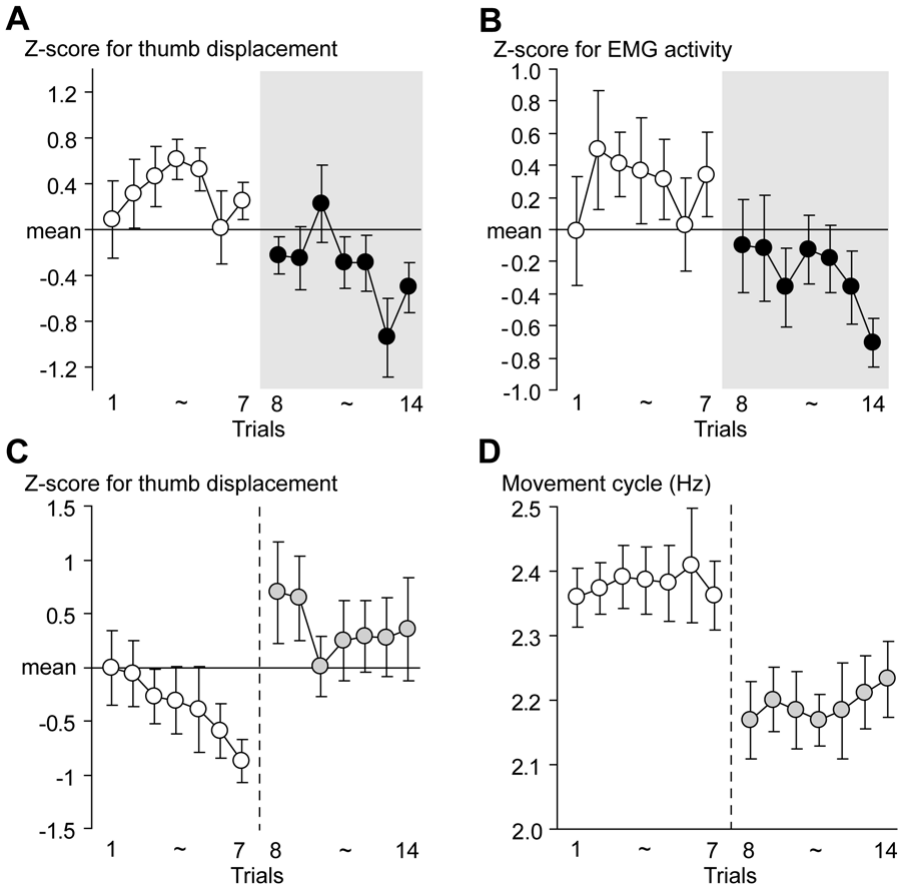
Results from control experiments. (A, B) Results from constant rotation task. (A) Average z-scores for amplitude of thumb displacement per cycle across participants (*y* axis) in sequence of trials (*x* axis). (B) Average z-scores for integrated EMG per cycle recorded from the APB muscle (*y* axis) in sequence of trials (*x* axis). White and black dots in both A and B panels represent data in NON-stimulation and stimulation trials, respectively. Each bar indicates SEM. (C, D) Results from maximum rotation task when we evaluated effect of volitional effort in motor performance. (C) Average z-scores for amplitude of thumb displacement per cycle across participants (*y* axis) in sequence of trials (*x* axis). (D) Average movement cycle across participants (*y* axis) in sequence of trials (*x* axis). White and gray dots in the C and D panels represent data in trials under unspecified and specified instructions about motor strategy, respectively.

In the constant rotation task, similar to the maximum rotation task, most participants reported that their movements became smoother in the stimulation trials. The average movement cycle across participants was 1.8 Hz, which was consistent between the NON-stimulation and stimulation trials. Yet we found significant reduction of amplitude of thumb displacement per cycle [F(1, 11) = 7.5, p<0.05] and its associated reduction of EMG activity [F(1, 11) = 5.7, p<0.05] in the stimulation trials, which were not noticed by the participants (Figures 2A, B). Thus, the reduction of finger displacement was a genuine behavioral effect from the thenar stimulation, irrespective of whether the task required maximum rotation or constant rotation at the regular pace where no particular motivation to exert maximum effort was necessary.

In the control experiment where we tested whether the participant's volitional effort of changing motor strategy (reducing amplitude of finger displacement) improves the stabilized motor performance, as did the thenar stimulation, all participants reported that the task became very difficult to perform after the 8th trial, where the motor strategy was specified. This difficulty seemed to confuse their motor control because the participants failed to reduce the amplitude of finger displacement; rather the amplitude tended to become greater in these trials despite the instruction (Figure 2C). Eventually, the immediate strategic change decreased the movement cycle (Figure 2D; from 2.4 Hz on average for the first seven trials to 2.2 Hz after the 8th trial). Two-way ANOVA revealed that the volitional effort worsened motor performance [F(1, 6) = 19.4, p<0.01]. This finding strongly supported the view that the participant's effort to intentionally change motor strategy could not help in improving the performance. Thus, it was externally administered thenar stimulation that helped reducing amplitude of finger displacement in stimulation trials of the maximum rotation task, which was associated with the performance improvement (Figure 1E).

These findings corroborated the view that the thenar stimulation reduced redundant motor components that could not essentially contribute to the ball rotation, which was difficult to achieve through the participant's volition.

### Experiment 3: Daily repetition of training with thenar stimulation facilitated motor learning beyond the plateau

**Figure 3:**
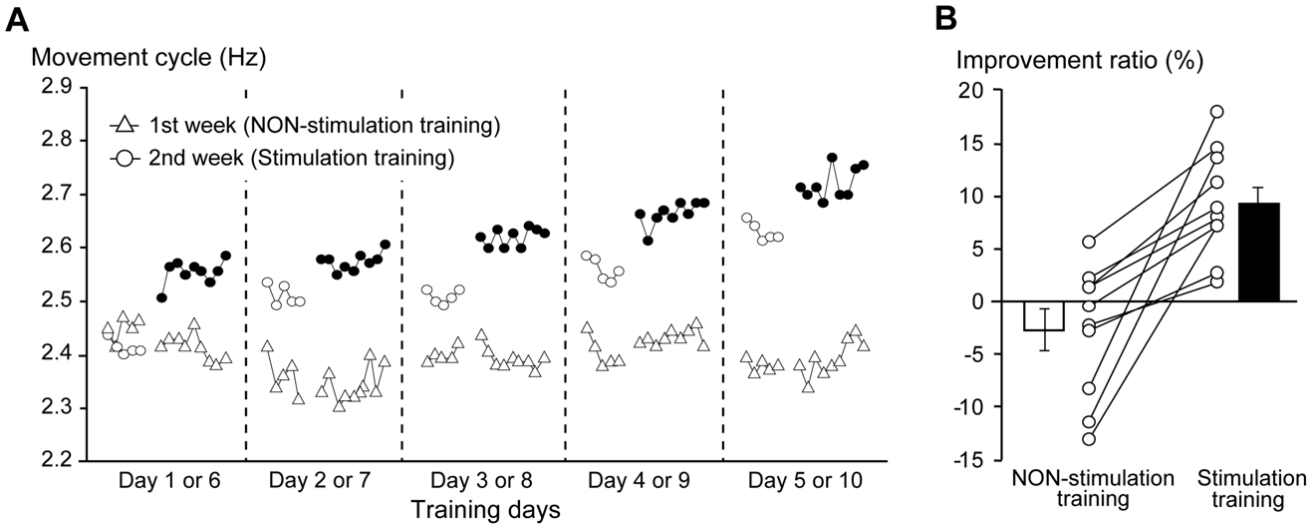
Results from two-week training. (A) Average movement cycle across participants (*y* axis) in sequence of trials in each training day (*x* axis). Triangles represent the movement cycle of each trial during NON-stimulation training. White dots represent the cycle of 1st-5th trials (NON-stimulation trials), and black dots represent those from the 6th–14th trials (stimulation trials) during stimulation training. (B) Improvement ratio of movement cycle in baseline performance (1st-5th) on the last (5th or 10th) day in each week compared to that on the first (1st or 6th) day of the corresponding week. White and black bars indicate the average improvement ratio across participants in each week. Each bar indicates SEM. A pair of dots connected by a line represents the improvement ratio of each participant. Left-side dot is for the first week (NON-stimulation training) and right-sided for the second week (stimulation training).

In the NON-stimulation training (1st-5th days), even though the participants were instructed to exert maximum effort, the performance never systematically improved spontaneously within the trials in each experimental day (Figure 3A), which was consistently observed across the days. Likewise, no daily improvement was achieved across the NON-stimulation training days. Indeed, the improvement ratio in the 1st-5th trials of the final (5th) day compared to the first day was −2.8% on average across participants (Figure 3B). Thus, the performance seemed to plateau throughout the NON-stimulation training days (Figure 3A).

In the first day of stimulation training (6th day), the baseline performance in the 1st-5th NON-stimulation trials was almost at the same level as that observed during the NON-stimulation training. This indicated that experience of motor training in the first week did not affect the baseline performance on the first day of the second week. The performance improvement was, however, possible in the 6th–14th stimulation trials on that day, which clearly indicated the stimulation effect. Performance immediately improved in the first stimulation trial (fast-acting effect), and the improved performance held during repetition of stimulation trials (retention; Figure 3A). Importantly, this was consistently observed on each day of the stimulation training (6th–10th days). These stimulation-dependent behavioral changes refuted the view that natural motor learning was somehow spontaneously facilitated in the second week. Three-way ANOVA showed that the performance improvement in the 6th–14th trials was only observed during stimulation training [F(1, 9) = 26.0, p<0.001; Figure 3A]. The same ANOVA also revealed that daily performance improvement was lopsided during stimulation training days [F(4, 36) = 6.5, p<0.001]. Indeed, during stimulation training, we found that the improved effect in one day partially carried over to the next day, thereby advancing the baseline performance in the 1st-5th trials on that day with no aid from the thenar stimulation. Eventually, the performance in the 1st-5th NON-stimulation trials improved daily during stimulation training, which was not found during the NON-stimulation training [F(4, 36) = 6.9, p<0.001; Figure 3A]. The improvement ratio of the baseline performance in the 1st-5th trials of the final (10th) day compared to the first (6th) day was 9.3% on average across participants (Figure 3B). The improvement in the baseline performance was greater during stimulation training in all participants, compared to that during NON-stimulation training. Thus, daily repetition of training with thenar stimulation promoted daily improvement of plateaued performance, which could not be systematically unlocked with one-week intensive training without thenar stimulation.

## Discussion

The performance improvement (increase of movement cycle) was only possible in the thenar stimulation trials but not in the wrist stimulation trials. Thus, the thenar stimulation was effective, and involvement of attentional factors in the performance improvement should be refuted, as the wrist stimulation also likely attracted the participant's attention to the hand similar to with the thenar stimulation. We also found no performance improvement in the sham stimulation trials. This indicates that either “effective” stimulation requires certain period of stimulation time or motor performance should be initiated immediately after the cessation of stimulation in order to get beneficial effects from the stimulation, or both. But, at least, this result has ruled out the possibility that the beneficial effect from the thenar stimulation is merely attributed to placebo effects from the stimulation, i.e. participants expect something good happens after the stimulation.

The behavioral changes in the thenar stimulation trials could be attributed to the net effect of electrical stimulation as somatic stimulation (see below), though the exact mechanisms of how this stimulation allowed the nervous system to improve the performance need to be clarified. This claim is supported by different evidence. First, the performance never improved even when the participants exerted their maximum effort within an experimental day (Figure 1B) and even across the days (Figure 3A), but the stabilized performance was improved by the application of the stimulation with the explicit subjective experience of smooth performance (Figure 1C). Second, the stimulation reduced the amplitude of finger displacement while the participants did not notice this change, which was observed even during the constant rotation at the regular pace where no particular motivation to exert maximum effort was required (Figure 2A). Finally, even when the participants tried to switch to the motor strategy (reducing amplitude of finger displacement) that was essential for performance improvement, this was very difficult to perform (Figure 2C) and the volitional effort never improved, but rather worsened, the performance (Figure 2D). All support the view that the performance improvement was unlikely attributed to other possibilities such as changes in volitional effort (motivation) and motor strategy.

The reduction in amplitude of finger displacement appeared to be a genuine behavioral outcome from the stimulation effect, as this was consistently observed irrespective of the task demands (Figures 1C, 2A). The reduction of amplitude of finger displacement accompanied reduction of its associated muscular activity (Figures 2A, B), which was generally observed in association with performance improvement in this task [6]. Since reduction of amplitude of finger displacement was also associated with performance improvement in the present study (Figure 1E), this change must be essential for goal-directed optimal control of the task.

The present short-period stimulation most likely modulated and remodeled sensory-motor neuronal states in a non-task-specific way [15], presumably in a distinct way when using long-period stimulation that can produce long-term potentiation (LTP)-like modulation in the central motor system [16], [17]. As described, high-frequency electrical stimulation is capable of modulating the sensory-motor neuronal states so as to generate a non-task-specific effect of reducing excessive motor activity, such as muscle hypertonia (see introduction). In this way, we found that the present stimulation could reduce redundant motor activities that are not essentially required for execution of the performance. Thus, the neuronal states likely modulated by this stimulation might be the key for the nervous system to unconsciously promote efficient control of subsequent motor performance. This indicates the potential effectiveness of short-period, high-frequency electrical stimulation. The general effectiveness of short-period somatic stimulation has been recently demonstrated: high-frequency electrical afferent stimulation in the spinal cord, even if it is short-period (less than one minute), is capable of modulating neuronal discharge in the cortico-subcortical motor circuit, which can facilitate permissive motor behavior of animals [18].

In the present trained participants, without stimulation, their performance never improved, even when repeating ordinary physical training across five days. In contrast, the performance improved in association with the stimulation consistently throughout the stimulation training days. This suggests the effectiveness and consistency of the thenar stimulation effect at least in the present participants and task. Even though we could not show the change of amplitude of finger displacement across days (see methods), based on the relationship between the performance improvement and the kinematic amplitude of finger displacement (Figure 1E), we presume that the kinematic change was somehow associated with the performance improvement across days (Figures 3A). Simple repetition of physical training of the stabilized motor skill might merely reinforce habitual access to its routine neuronal circuits, thereby resulting in no substantial performance improvement across the days. In contrast, the sensory-motor remodeling by the stimulation might assist the nervous system in shifting to a further optimal state, which allows upgraded performance. Surprisingly, this shift was possible even in the very first stimulation trial (fast-acting effect; Figures 1B, C), and its stabilization could be established through repetition of stimulation trials (retention; Figures 1B, C) where the upgraded control processes might be consolidated in the nervous system. This type of neuronal operation must be necessary to generate the carry-over effect in the next day's performance (Figure 3A), which promoted motor skill learning beyond the plateau. We speculate that the daily skill improvement (Figure 3A) was mediated by long-term plastic change in the central motor system as previously reported [19].

In summary, electrical somatic stimulation allowed not only for upgrading control of the plateaued motor skill but also for promoting motor skill learning at the plateau stage. Thus, this study raised the possibility that a human plateaued motor skill can be effectively improved with pre-movement somatic stimulation, which is available for anyone at any time, and in any place.

## Supporting Information

Figure S1 **Stabilized motor performance in each participant before the experiments started.** Movement cycle (*y* axis) in sequence of trials (*x* axis). White dots represent data from each participant. Gray squares represent average movement cycle across participants.
(TIF)

Text S1 **Evidence of behavioral plateau in each participant.**
(DOC)
