# Supplementary figures and images for: Improving Human Plateaued Motor Skill with Somatic Stimulation
Source: PLoS One. 2011 Oct 4;6(10):e25670. doi: 10.1371/journal.pone.0025670 (PMC3186792; doi:10.1371/journal.pone.0025670)

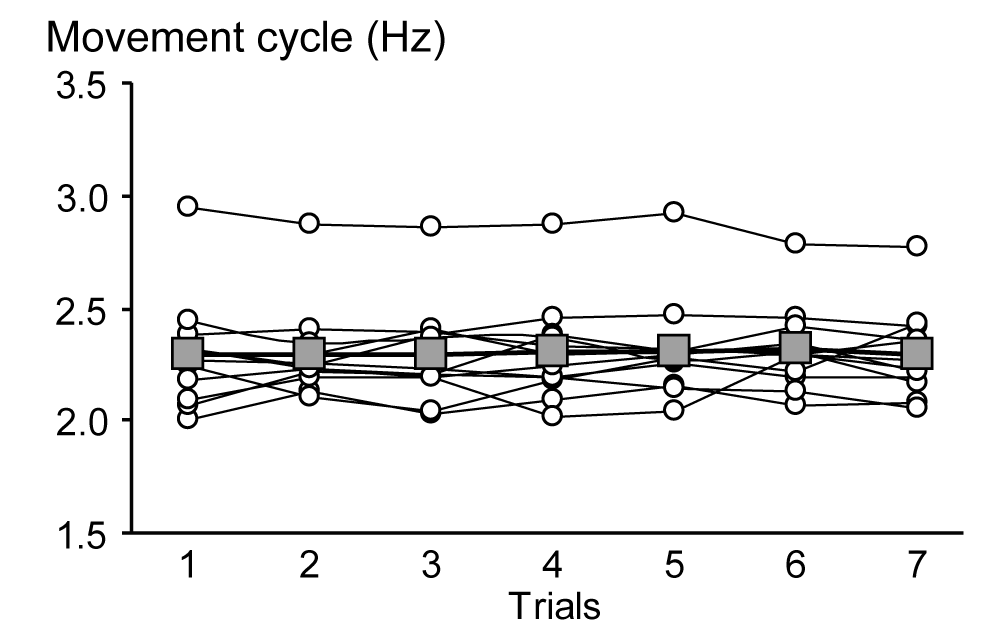

Supplement: Figure S1 — Stabilized motor performance in each participant before the experiments started. Movement cycle (y axis) in sequence of trials (x axis). White dots represent data from each participant. Gray squares represent average movement cycle across participants. (TIF) [file pone.0025670.s001.tif]
