# Supplementary material for: Improving Human Plateaued Motor Skill with Somatic Stimulation
Source: PLoS One. 2011 Oct 4;6(10):e25670. doi: 10.1371/journal.pone.0025670 (PMC3186792; doi:10.1371/journal.pone.0025670)
Supplement: Text S1 — Evidence of behavioral plateau in each participant. (DOC) [file pone.0025670.s002.doc]

**Text S1. Evidence of behavioral plateau in each participant.** Before we started the first experiment, we confirmed that all (12) participants could rotate the balls at their stabilized cycle with no significant improvement within trials. In this investigation, the participants performed the same maximum rotation task (seven 15-s trials with ITI of 50 s). The movement cycle in each trial in each participant is shown in supporting Figure S1. In each participant, we calculated the average improvement ratio between trials, and performed one-sample t-test to see if the improvement ratio was significantly greater than zero. We found no significant improvement in any of participants. Thus, the participant’s performance was stabilized before the experiments, which could be safe to say that their performance had reached the plateau stage.
